# Mental Side Effects of Deep Brain Stimulation (DBS) for Movement Disorders: The Futility of Denial

**DOI:** 10.3389/fnint.2016.00017

**Published:** 2016-04-20

**Authors:** Donatus Cyron

**Affiliations:** Neurosurgery, Städtisches Klinikum KarlsruheKarlsruhe, Germany

**Keywords:** deep brain stimulation, psychiatric side effects, suicide, moral competence, depression, Parkinson's disease

The benefits of deep brain stimulation for parkinsonian patients are well documented and have established the method as mainstay in the late stages of the disease (Deuschl et al., 2006). However, early in the history of the method reports of mental side effects were published. In 1995 Limousin and colleagues reported transient confusion and hallucinations with one of their first patients (Limousin et al., 1995). Further reports with disturbing side effects accumulated over time (Krack et al., 2001; Berney et al., 2002; Herzog et al., 2003a). While cognitive squeals were studied in numerous papers (Funkiewiez et al., 2004; Contarino et al., 2007) but are generally conceived to have little impact on the quality of life (Schupbach et al., 2006), the field of psychiatric effects was more hesitantly explored. Results to the latter remain ambiguous with a tendency toward less severe side effects in the large series of experienced centers (Deuschl et al., 2006; Weaver et al., 2009). Adverse events in this domain were largely attributed to acute effects weaning over a short period (Herzog et al., 2003b).

However, over the years I have seen a considerable number of patients both operated by myself but also from other experienced centers who showed psychological symptoms that counteracted the improvements in motor function. This personal experience is in line with patients and their representatives who voice concern over these phenomena with their deleterious impact on the wellbeing of patients, their families and caregivers (personal communication, F-W. Mehrhoff, Geschäftsführer, Deutsche Parkinsonvereinigung, Nov. 2014). Such concerns also appear quite regularly in patient support group meetings. My impression is, that patients and their representatives feel their concerns not appropriately reflected in the scientific literature and expert opinions. This comment responds to such observations with the aim to reposition the defense of DBS for Parkinson's disease. Reluctance to delve into this subject may in my opinion eventually leave those who offer the procedure defenseless toward reproach from patients, referring colleagues and the general public.

One may counter such observations by outlining that, particularly in case of complex psychiatric side effects, there are no objective means of deciding when a treatment has to be considered a failure or a success. Patients and relatives have also the propensity to underestimate their preoperative disabilities (Herzog et al., 2003b). This is further complicated by the fact that PD is a disease with cognitive, affective, and behavioral symptoms and thus has a neuropsychiatric impact on the patient as well. DBS may even in some cases restore the original personality, which then is simply not fitting any more into the actual social and familial setting. Finally, also medication-based therapies for PD can have severe neuropsychiatric effects (Cools et al., 2003).

Nevertheless, there remains a substantial proportion of DBS patients with severe and lasting behavior disturbances, which were credibly not present in the ultimate preoperative phase and that also has led to critical comments in the literature (Moro, 2009). These comprise reckless driving or other forms of risk-seeking behavior and even aggressive and contemptuous behavior toward relatives and spouses. In some cases this can be remedied by moving the active contacts outside the STN with subsidence of aggressive behavior within hours. This hints to a direct effect of stimulation. Unfortunately in some patients stimulation must be markedly reduced or switched off completely to ameliorate psychiatric effects. Numerous reports and case series have contributed to this issue with delineation of alarming psychiatric disturbances ranging from hypomania to suicidal ideation and suicide (Herzog et al., 2003a; Morgan et al., 2006; Schupbach et al., 2006; Witt et al., 2008). At the same time other publications failed to find relevant changes of personality and behavior (Schuepbach et al., 2013), considered significant changes as not relevant for overall quality of life (Morgan et al., 2006) or even found much higher incidence of psychoses in the control group compared to the DBS group. I will now elaborate on these issues in some more detail.

## Mood and behavior

A symptom that may be systematically underestimated in its frequency is hypomania. It occurs early after the intervention and may lead to apparent improvement in rating scales like the Beck Depression Inventory and scales measuring quality of life. The subjective rating by the patient may however be a result of direct stimulation of the limbic system and not a normal joyful response to the improved motor performance. Hypomania becomes dangerous though impaired judgment of the own abilities and limits. While the patient (and the surgeon) may be content or even happy with the result, the patient's family may be dismayed about disinhibited and reckless behavior.

After some weeks hypomania may subside but other alterations in mood and behavior surface. Depression and Apathy may evolve and stay for years leading to markedly reduced quality of life not only for the patient but also for his caregivers. The reasons for both remain unclear and have been attributed to the stimulation directly or the resulting changes in medication. The incidence of both is relatively well reflected in the scientific literature (Drapier et al., 2006) with a minority finding contradicting results. With few exceptions the burden resulting from altered mood and behavior on caregivers and families has not received much attention (Lewis et al., 2015).

## Suicide

While some studies found markedly elevated incidences of suicide (Voon et al., 2008) a direct association with DBS surgery was not found in a recent prospective study (Weintraub et al., 2013). This may indicate improvements regarding selection criteria—but the observation time in latter study was however short and patients were in the close scrutiny of a prospective setting allowing early detection and intervention if suicidal ideation occurred. The issue is further complicated by the fact that suicide after DBS has occurred not only with different anatomic targets but also with other diseases than Parkinson's disease (Appleby et al., 2007). Rare events like suicide are statistically difficult to assess, however, it is alarming that even in prospective studies with close monitoring of patients these events occur (Schuepbach et al., 2013). From a laymen's viewpoint the situation remains confusing; patients and their relatives remain worried due to reports of such events in patient representative organizations that seem to be in contrast to studies and expert opinions. To my understanding, a definite proof of the safety of the procedure in this respect is at present not available and the matter may eventually only be solved by a comprehensive DBS case registry.

## Moral competence and personality

These are (in my view) the issues with the most confounding ethical impact. While hypomania subsides and depression may respond to treatment, rarely measurements are taken to exclude less obvious changes in behavior and personality. Fundamental changes in personality have been relatively seldom been studied (Florin et al., 2013) although basic research documents the role of the STN in decision making (Ray et al., 2011). Relatives however report reckless and risk seeking behavior that lasts well beyond the postoperative phase. Again, these types of behavioral change may occur gradually in the natural course of the disease or as side effect of drugs—but the sudden and seemingly irreversible alteration of personally after DBS comes as a shock for families. Probably the most profound, albeit at first sight easily overlooked effects are changes in moral competences of the patients. Such changes may result in taking risks for oneself and ignoring the rights of others, exemplified by car accidents or marital conflicts (Schupbach et al., 2006). Seminars for management of such conflicts after DBS are therefore already offered with patients and spouses reporting their experiences.

Changes in personality or moral competences have so far not been an issue in recent large studies (Schuepbach et al., 2013). One reason might be that they are difficult to measure with the scales presently at hand. Scales using the patient's own perception are fated to miss deficits in his ability to cope with the needs of others. Recently, the issue has come into focus (Fumagalli et al., 2015), but more appropriate scales to detect and quantify such complex changes must be implemented in the future (Witt et al., 2013). Cooperation with specialists in the area of moral psychology should be considered to adopt scales and knowledge.

## The STN as target

One reason for the frequency of psychiatric side effects may be the stimulated target. As the standard target for DBS in Parkinson disease the STN must be foremost scrutinized.

One of the basic assumptions in defense of the safety of the STN is the assumption that it consists of three clearly separated parts and that electrodes can be reliably steered into the motor part that exclusively contains motor functions. This concept while propagated by some researchers (Temel et al., 2005) has at the same time been questioned by others (Keuken et al., 2012). Furthermore, with the assumed motor division being quite small currents from the presently used electrodes will almost inevitably spread to other parts namely the limbic subdivision (taking into account that currents spread sideways from the electrode into areas outside the intended target).

Empirically the majority of studies come to the conclusion that targeting the GPI produces less psychiatric side effects (Liu et al., 2014) while allowing for almost equal motor improvement. Only one recent study found a clear advantage for the STN (Odekerken et al., 2013). Disadvantages of GPI are a smaller reduction of medication and possibly a weaning of effect over time. The necessity to keep up a high level of dopaminergic medication may itself provoke psychiatric events (Volkmann et al., 2004). Still the GPI may serve as target for a subset of patients prone to complications by STN-stimulation as outlined in a recent comment (Krack and Hariz, 2013). As such it has been advertised as target for patients prone to psychiatric complications^1^.

The evaluation search for alternative targets should therefore be continued. Other promising targets are located in the subthalamic space within the fiber connections between thalamus cerebellum and basal ganglia as proposed by Velasco and others. However, it must been kept in mind that the basal ganglia and the cerebellum are involved in the processing of cognitive and emotional tasks and with the effects of stimulation spreading far complete avoidance of mental side effects is therefore not possible.

## Perspectives

In summary, the available evidence suggests to my understanding that neuropsychiatric effects may be an integral albeit unintended effect of DBS for Parkinson's diseases. The ethical issues related to these effects may be even less trivial compared with DBS for psychiatric disorders. In the latter case, DBS is used with a transparent and obvious intention to interfere with the patient's psyche (Chabardes et al., 2013). In DBS for Parkinson's disease, the neuropsychiatric effects are not an obvious “part of the deal” with the patient.

Subsequently a sober and unprejudiced discussion about how far these phenomena can be accepted should evolve. For fear of putting a well-established and beneficial procedure at peril, its disadvantages shall not be disguised.

The discussion about target points that carry a lower risk of changing the patient's personality should continue. Patients must be informed about the available targets and the risks and benefits connected with each of them.

Prerequisite for the future development of DBS are diagnostic tools that are reliably able to detect the long-term consequences of the operation on behavior, personality and moral competence. Suitable scales can be developed or integrated from other disciplines into the clinical assessment and future studies. These must concentrate on the crucial domains of social interaction and judgment of own behavior. They should also address caregivers and family's perception of the patient and the impact on their life.

In defense of the procedure a reversal in the argumentation should be considered:

- The existence of psychiatric side effects as integral part of the treatment should be conceded.
- It should be asserted that DBS is a reversible therapy and can therefore not be put on a par with irreversible surgical interventions like the lesioning of brain areas. In analogy to drug therapy it can be discontinued simply by switching it off.
- The patient should be fully informed about the intrinsic risks of psychiatric adverse events, possible changes of personality and the targets at hand. Ideally the patient's partners and next of kin are involved and informed about the possible resulting burden as caregivers. This will counter the reproach of guiding patients into a treatment with consequences he cannot foresee.

Clearly the issue of psychiatric side effects of DBS involves a legally and ethically utterly sophisticated discussion. Yet postponing it beyond the moment the lay press may cover negative events in an untoward manner will not improve our position to influence its already unforeseeable outcome.

## Author contributions

The author confirms being the sole contributor of this work and approved it for publication.

### Conflict of interest statement

The author declares that the research was conducted in the absence of any commercial or financial relationships that could be construed as a potential conflict of interest. The author has received grants for contributing to the development of stereotactic planning systems from Medtronic and Precisis.

## Footnotes

^1^Radio broadcast in Deutschlandfunk covering the work of a implantation center in Germany www.dradio.de/dlf/sendungen/forschak/1407092/

## Abbreviations

DBS: Deep brain stimulation
GPI: Globus pallidus internus
STN: Subthalamic nucleus.
